# Obesity prevalence in a cohort of women in early pregnancy from a neighbourhood perspective

**DOI:** 10.1186/1471-2393-9-37

**Published:** 2009-08-25

**Authors:** Eva Sellström, Göran Arnoldsson, Marie Alricsson, Anders Hjern

**Affiliations:** 1 Department of Health Sciences, MidSweden University, Östersund, Sweden; 2 Department of Statistics, Umeå University, Umeå, Sweden; 3 The Vårdal Institute, The Swedish Institute for Health Sciences, Lund University, Lund, Sweden; 4 Centre for Epidemiology, Swedish National Board on Health and Welfare, Stockholm, Sweden; 5 Centre for Health Equity Studies, Karolinska Institute, Stockholm, Sweden

## Abstract

**Background:**

The evidence of an association between neighbourhood deprivation and overweight is established for different populations. However no previous studies on neighbourhood variations in obesity in pregnant women were found. In this study we aimed to determine whether obesity during early pregnancy varied by neighbourhood economic status.

**Methods:**

A register based study on 94,323 primiparous pregnant women in 586 Swedish neighbourhoods during the years 1992-2001. Multilevel technique was used to regress obesity prevalence on socioeconomic individual-level variables and the neighbourhood economic status. Five hundred and eighty-six neighbourhoods in the three major cities of Sweden, Stockholm, Göteborg and Malmö, during 1992-2001, were included. The majority of neighbourhoods had a population of 4 000-10 000 inhabitants.

**Results:**

Seven per cent of the variation in obesity prevalence was at the neighbourhood level and the odds of being obese were almost doubled in poor areas.

**Conclusion:**

Our findings supports a community approach in the prevention of obesity in general and thus also in pregnant women.

## Background

Overweight is a predominant health problem worldwide and is today regarded as one of the most important health threats [1]. This epidemic is especially pronounced in young people, including women of reproductive age. A Swedish study reported a six-fold increase since 1980-81 in obesity prevalence in women 16 to 34 years of age [2]. Further, an overview by Guelinckx et al reported obesity prevalence in pregnant women from different countries ranging from 1.8% to 25.3% [3]. Four recent systematic reviews summarise foetal and maternal negative consequences of obesity during pregnancy [3-6]. Maternal risks include gestational diabetes, hypertension and pre-eclampsia, increased incidence of operative delivery, postpartum haemorrhage, and anaesthetic risks, as well as infective and thromboembolic complications, while foetal risks include miscarriage, neural tube defects, macrosomia, and stillbirth.

As a consequence, a number of intervention programmes to prevent or control obesity during pregnancy have been developed, and several of these programmes have adopted a community approach [6]. Even if no conclusive evidence of the effects is currently at hand, the community approach implicates a more narrow focus on the close environment. Accordingly, an increased interest in how neighbourhood environments influence the risk of being overweight is apparent in the literature. Neighbourhood characteristics that possibly influence obesity prevalence have thus been investigated. The evidence of an association between neighbourhood deprivation and overweight is established for different populations [7-15]. However, we found no previous studies on neighbourhood variations in obesity in pregnant women. In the present study we investigated neighbourhood variations in obesity prevalence in Swedish primiparous women in their early pregnancy. The study is based on data from the Medical Birth Register where the weight and height of women are measured at their first visit at the primary care clinic. We hypothesise that there is an association between neighbourhood deprivation and obesity in pregnant women, and that the trend of obesity in pregnancy has increased during the time period of observation.

## Methods

### Design and variables

All primiparous pregnant women seeking maternity care in the three major cities in Sweden, Stockholm, Göteborg and Malmö during the years 1992-2001 were included in the study. Thus, the study was based on register information regarding the pregnant women linked through each individual's unique Civic Registration Number [16-19]. Data on pregnancies were collected from the Swedish Medical Birth Register held by the National Board on Health and Welfare in Sweden, at the first antenatal visit at the maternity care during the years 1992-2001 [17,19]. Socioeconomic individual-level variables came from the LOUISE-database held by Statistics Sweden and was gathered two years before the year of birth of the infant (1990-1999) [18,19]. Neighbourhood economic status, i.e. an aggregated measure on income, was included as a second-level variable [19,20]. Data on neighbourhood economic status was collected on the 31 of December of the year prior to the birth of the offspring of the reported pregnancy (1991-2000). The study was approved by the Ethics Committee of Umeå, Sweden.

The cities were originally divided into 696 neighbourhoods; the majority of neighbourhoods have a population of 4,000-10,000 inhabitants. Neighbourhoods with fewer than 500 inhabitants were excluded. The original data set included 142,852 primiparous women with singleton pregnancies. Teenage pregnant women (n = 4,080; 2.9%) were excluded from this analysis since the socio-demographic variables available in the registers referred to their parents. Women where information on height and weight was lacking or obviously wrong were also excluded (n = 27,804; 21.6%). Cases with no information on neighbourhood economic status were excluded (n = 722; 0.7%). Finally, in order to avoid bias, we excluded foreign-born women as there is evidence of a biologically grounded association between ethnic origin and risk of overweight [21,22]. Thus, 94,323 women remained as participants in the study.

### Neighbourhood-level variables

Areas in the region were defined as neighbourhoods by natural geographic borders and homogeneity of housing [20]. The aggregation of neighbourhoods was based on a system of geographical coding in which all estates in Sweden obtain a key code [20,23]. In this study we used an aggregated measure relating to each neighbourhoods' economic status [20]. This variable was created for each year of the study. Thus, a neighbourhood has a unique code for each year and the code may differ depending on the year of observation.

'Economic status' was calculated as the ratio of low-to high-income earners in the neighbourhood (LH-ratio). Income earners are divided into three classes, low, normal and high. Low income earners are defined as belonging to the lowest quintile of income earners in the actual regions; Stockholm, Malmö, Göteborg. In 1990 earners with an income below 123,300 SEK (Stockholm), 114,500 SEK (Malmö) and 123,800 SEK (Göteborg) were defined as low income earners. In 2002 the corresponding limits for low income earners were 151,600 SEK, 121,600 SEK, 150,200 SEK. High income earners are defined as belonging to the highest quintile of income earners in the region. In 1990 earners with an income above 255,400 SEK, 225,300 SEK and 232,500 SEK respectively were defined as high income earners. In 2002 corresponding limits for high income earners were 406,800 SEK, 352,400 SEK, 364,600 SEK. Thus, the classification of neighbourhood economic status is based on a continuous measure, trichotomized for the purpose of this study. The three categories were ranging from homogeneously affluent areas to homogeneously poor areas and coded as 1 = affluent; 2 = medium level of resources; 3 = poor.

The number of neighbourhoods varies between years as only neighbourhoods with registered pregnancies were included. During the period observed the character of the neighbourhoods changed towards fewer neighbourhoods with a medium level income status. However, this change was not significant (chisquare 20.238, df 18, pvalue 0.32). In Table 1, we present the distribution of neighbourhoods for the each year of observation. Fewer neighbourhoods had a medium level economic status and more neighbourhoods were either classified as poor or affluent.

**Table 1:** Distribution of neighbourhoods regarding economic status.

**Neighbourhood economic status**	**1992**	**1993**	**1994**	**1995**	**1996**	**1997**	**1998**	**1999**	**2000**	**2001**
Affluent	57	60	69	65	65	67	74	77	78	80
Medium level	398	393	368	378	375	371	357	357	354	353
Poor	122	120	137	133	139	139	141	142	143	153
Total	577	573	574	576	579	577	572	576	575	586

The rationale for using this ratio is that not only the prevalence of low income earners in an area but also of high income earners plays an important role for the neighbourhood social climate. Having a high income coincides for example with better education and high income households can be assumed to have a strong demand on public and commercial services of good quality, such as schools, primary care etc in their neighbourhood. Accordingly, the presence of high income households constitutes a stabilizing factor within the neighbourhood and as such is beneficial to the whole area.

### Body mass index (BMI)

As a measure of relative weight, BMI is easy to obtain (weight in kilos divided by height in square metres, kg/m^2^). Based on World Health Organisaton (WHO) guidelines, obesity is defined as BMI >30 [24]. In this study a dichotomous outcome (coded as 0 = non-obese; and 1 = obese) was used. We assessed different outcome classification schemes. At first, a four category variable was used; obese, overweight, normal weight and under weight. We also assessed a dichotomous variable where the first category was obese or over weight women and the other category was normal or under weight women (data not shown). Significant difference was found between obese subjects compared to all other subjects, and the dichotomized obese/not obese categorization was used as outcome in the analyses presented in the paper.

Information on primiparous pregnant women's weight and height is included in the Medical Birth Register and was collected at the first antenatal visit to the maternity ward [17]. The most frequent time for an initial visit to an antenatal clinic was after ten full weeks of pregnancy. Roughly 90 per cent have made an initial visit after twelve full weeks. In the medical records sample, the date of the first visit had not been registered in four per cent of cases. The validation of the register found it possible to calculate BMI for about 65-86% of the women [25].

### Individual-level variables

Data on the women's age, and family status (coded as 0 = cohabiting; and 1 = single) were included. Smoking habits during pregnancy (coded as 0 = 0 cigarettes daily; 1 = one to nine cigarettes daily; 2 = ten or more cigarettes daily) were derived from the Medical Birth Register [17]. Data on the mothers' educational backgrounds were obtained from Sweden's Education Register, and coded as 4 = university; 3 = three to four years of secondary school; 2 = two years of secondary school; 1 = primary school [18]. Data on income refers to disposable income, calculated by summing up all incomes in the household, including societal benefits, deducted by income tax and divided by the size of the household according to a formula used by Statistics Sweden for this purpose. Income data was presented as quintiles, where the first quintile refers to the lowest income and the fifth to the highest.

### Statistical analyses

The MLwiN version 2.01 was used to perform the multilevel modelling [26]. Parameters in the random effects model were estimated using iterative generalized least squares. Analyses were performed using multilevel logistic regression technique [27,28]. The conceptual basis for a two-level model, as used in this study, is that the effect (e.g. the beta-coefficients in a regression) of individual-level variables, in this study: demographic factors and the women's SES differs between neighbourhoods (level 2). WALD test was used to test the statistical significance of parameters included in the models.

The first model was called a base model in which' year of observation' was entered as a fixed effects coefficient. The year variable was centred on the middle year, 1997, with a value of 0, the first year, 1992 with a value of -4.5 and the latest year, 2002, 4.5. The model can be expressed with the following formula:

(1)

To handle 'year of observation' as a fixed effect coefficient implies that the unknown variation between neighbourhoods was proportionately the same across all years. This restriction was tested by Wald statistics.

The second model is an individual level model and relates the log of P to individual-level variables:

(2)

The full multi-level model relates the individual-level intercept to neighbourhood-level variable, as follows:

(3)

where γ_1 _is the effect of the current neighbourhood economic status on the level-1 intercept; μ_j _represents a random variation among neighbourhoods.

The degree of resemblance between women from the same neighbourhood can be expressed by the variance partition coefficient (VPC). The VPC is the proportion of variance that is accounted for by the neighbourhood level. In binary response models no single VPC measure is available since the individual-level variance is a function of the mean. Snijders and Bosker have described a threshold model based on the assumption that the logistic model is cast in the form of a linear threshold model and there is an unobserved continuous variable underlying the binary response variable [28]. The following formula was used:

(4)

where  denotes the neighbourhood-level variance in the intercept and the logistic distribution for the individual residual implies a variance of π^2^/3 = 3.29.

We present variance components and reduction of variance to assess the fit of the models. Reduction of unexplained variance is calculated on the basis of variance components obtained in the modelling procedure.

## Results

In all, 95.3% of women (n = 89,851) were not obese according to WHO's criteria, while 4.7% (n = 4,463) were. In Table 2 we present descriptive statistics for the sample. Among the younger women, obesity was not significantly more prevalent compared to older age-women. (chi2 = 3.412; 1 df; p-value = 0.065). Regarding education an apparent association was evident, among low educated women obesity was more common (chi2 = 573.217; 3 df; p-value < 0.001). The income variable showed a similar pattern (chi2 = 201.241; 4 df; p-value < 0.001). The study could also establish that smoking was more common among obese women (chi2 = 159.063; 2 df; p-value < 0.001).

**Table 2:** Descriptive statistics of obese and non-obese subjects (n = 94314).

	**Non-obese**	**Obese**	**Chi-square statistics**
**Age**			
20-34 yrs	90.4%	89,6%	Chi2 = 3,412
> = 35 yrs	9,6%	10,4%	df = 1,
			P-value = 0,065
**Education**			
Elementary	8.1%	13.6%	Chi2 = 573.217
Secondary (2 yrs)	49.4%	60.7%	df = 3,
Secondary (3-4 yrs)	20.7%	14.9%	P-value < 0.001
University	21.8%	10.9%	
**Income**			
1^st ^quintile (lowest)	7.2%	8.9%	Chi2 = 201.241
2^nd ^quintile	9.8%	12.7%	df = 4,
3^rd ^quintile	18.2%	21.4%	P-value < 0.001
4^th ^quintile	34.4%	36.2%	
5^th ^quintile	30.4%	20.8%	
**Smoking habits**			
Non smoker	86.4%	80.1%	Chi2 = 159.063
<10 cig a day	9.4%	13.0%	df = 2,
>10 cig a day	4.2%	7.0%	P-value < 0.001

The number of neighbourhoods varied between years as only neighbourhoods with registered pregnancies were included. During the period observed the character of the neighbourhoods changed towards being more homogenous, i.e. poorer or richer. In Table 1, the distribution of neighbourhoods regarding economic status, for each year of observation (1992-2001) is presented.

**Figure 1:**
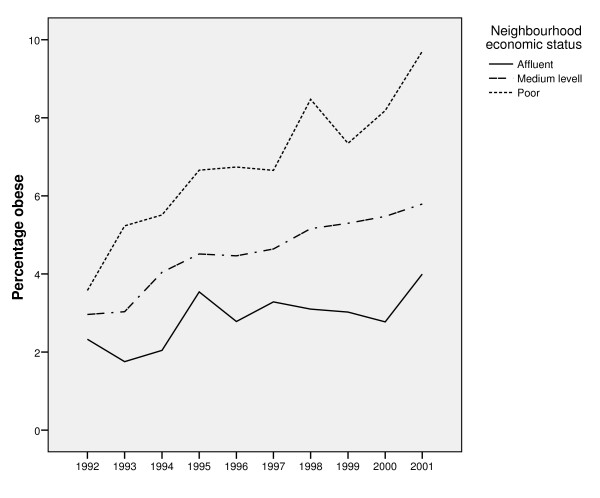
**Percentage obese women by neighbourhood economic status and year of observation**. The proportion of obese women by neighbourhood economic status during 1992-2001.

There was an increase in the proportion of obese women overall neighbourhoods during 1992-2001. However, this increase was more pronounced in the poorest neighbourhoods. In poor neighbourhoods, 3.6% of the women were obese 1993, i.e. the first year of observation, whereas in 2001 correspondingly 9.7% of the women were obese. See Figure 1.

In Additional file 1, the multilevel models are presented. First, a base model was elaborated (Model 1) with only 'year of observation' entered as a fixed effect coefficient. The odds of being obese increased by 8% per year during the 10-year observation period (OR = 1.08, 95%CI 1.07; 1.09). Using the threshold model, VPC was estimated to be 7.04%, i.e. 7.04% of variation in obesity prevalence was explained by the neighbourhood they lived in.

In Model 2, including only individual-level covariates, especially low maternal education (OR = 2.95, 95% CI 2.60; 3.36) constituted a strong individual-level predictor of obesity. Women age 35 or older were at higher risk of being obese compared to younger women (OR = 1.43, 95% CI 1.30;2,26). The income variable on the other hand, had a curvilinear association with the risk of obesity, indicating collinearity between individual-level covariates. This model reduced variance to 32.9%.

In Model 3, a neighbourhood model was elaborated. Women in poorer neighbourhoods were at higher risk of being obese. The odds of being obese increased by each category of neighbourhood economic status and in the poorest neighbourhoods the odds ratio were 2.25 (95% CI 1.91; 2.66). The neighbourhood model reduced the variance with 26.1%.

In model 4, the full multilevel model, which includes individual and neighbourhood variables, the strong association between neighbourhood economic status and the risk of being obese remained. In poor neighbourhoods the odds ratio were 1.80 (95% CI 1.54; 2.19). The variance component was reduced further and altogether 46.2% of variation was explained.

In this model, interactions were explored. Interaction between age and the woman's disposable income was not significant. Cross-level interaction between economic status of the neighbourhood and the woman's educational background did not either reach significance. Therefore interaction terms were not entered in the model.

## Discussion

In this study we can show that obesity prevalence in primiparous pregnant women has increased during a 10-year observation period. This increase was most pronounced in poor neighbourhoods. Compared to rich neighbourhoods the odds of being obese was 80 percent greater in poor neighbourhoods. These findings are supported by similar results in earlier studies. Van Lenthe and Mackenbach reported that odds ratios of overweight increased significantly by increasing neighbourhood deprivation [11]. Robert and Reither could show that living in communities with higher socioeconomic disadvantage was associated with higher body mass index (BMI) net of individual social risk factors [10]. Thus, the prevalence of obesity varies significantly with the economic status of the neighbourhood. This variation can be explained equally by individual and neighbourhood factors. Low education is a strong predictor for obesity in pregnant women which corresponds well with general findings in other Swedish samples [29-31]. However, 7 percent of the variation was at the neighbourhood level, indicating a potential for prevention [32].

There are certain limitations in the present study. Most women, 82.9%, come for their first antenatal visit early in their pregnancy, that is, before the 12^th ^week In a normal pregnancy the woman will already have gained some weight, but in cases of hyperemesis her weight will be abnormally lower. This means that some women will exceed a BMI > 30 due to the few kilos of weight gain, and some other women who have lost weight will not be registered as obese. According to a validation report on the Medical Birth Register measurements of weight at that time has a good quality [25]. Further, foreign-born pregnant women were excluded in order to avoid confounding the results. Foreign-born women are at higher risk of being obese and are also overrepresented in poor neighbourhoods [21,22,33]. Inclusion of these women would lead to an overestimation of the neighbourhood effect. Therefore we consider our results representative of Swedish-born primiparous pregnant women in their first trimester.

Information on BMI was lacking in 21.9% of the women, and these women were excluded from the analyses. It is well-known among practioners that obese women are reluctant to be weighed at their first antenatal visit, and therefore obesity prevalence is likely to be underestimated. If this under-reporting varied systematically between neighbourhoods, our results could be biased. We compared records where information on BMI was lacking to those where such data were available. The differences between respondents and non-respondents regarding individual social characteristics and neighbourhood prevalence were minor, and bias due to under-reporting is considered less likely.

We investigated different outcome measures. In this paper we present analyses of a dichotomous outcome, obese versus non-obese subjects. The rationale for this is two-fold. Firstly, obesity is the condition evidently negative to foetal and maternal health [3-6]. There is no strong evidence of a similar risk concerning over weight pregnant women. Secondly, studies on neighbourhood variations have mainly focussed on obese versus non obese subjects in different population groups [2,29,30]. Thus, in order to facilitate comparison we chose the dichotomous outcome; obese - non obese.

Controlling for the women's own income could possibly imply overcontrol of confounders. On the other hand, not controlling for individual income could mean that observed neighbourhood effect is due to unmeasured individual income variations. We therefore included the individual income variable in the multilevel model.

A strength of our study lies in the use of objective measures of weight and height of the women. Most studies on BMI are based on self-reports, and such data can be biased. Kuskowska-Wolk et al demonstrated the systematic tendency for overweight and obese subjects to underestimate their body size in self-reported data [34].

This study is based on register data for 10 years of observation, for which exposure data preceded the outcome under study. Data on neighbourhoods were collected the year prior to the year of each woman being included in the study. Many couples move to another area once there child is born or already during pregnancy. However, our results refer to primiparous women in early pregnancy and we can assume neighbourhood residency to be fairly stable, i.e. the women having lived in the same area for a longer period of time. These exposure data can therefore be considered as valid and support a causal interpretation of the observed associations.

The construction of neighbourhoods used in this study is based on a definition of a neighbourhood as a social arena for social identification and the symbolic value that the neighbourhood represents. The neighbourhoods are formed along natural geographic borders, with homogeneity of housing. The size of neighbourhoods used in this study, with ca 4000-9000 inhabitants, is larger compared to US census tracts containing an average of 4000 [35]. Large-size neighbourhoods imply a greater heterogeneity within the neighbourhood and dilute any effect of neighbourhood characteristics on the outcome under study. Thus, neighbourhood effects observed here could in fact be even stronger.

Variation in obesity prevalence between neighbourhoods can be interpreted as determined by contextual factors; that is, the structure of the neighbourhood mediates the association between the economic status of the neighbourhood and the risk of being obese. Several recent publications support such an interpretation. More fast food restaurants, fewer grocery stores, and low availability of recreational resources are all contextual factors shown to be associated with low economic status of the neighbourhood [36-41]. Similarly, collective efficacy, exercise, and physical activities were less prevalent among citizens in low income neighbourhoods [7,42].

## Conclusion

This is, to our knowledge, the first study on neighbourhood variations in obesity in pregnant women. Our results show a substantial variation between neighbourhoods and the odds of being obese were almost doubled in poor areas. These findings are important since they indicate a community approach in the prevention of obesity in pregnant women. Even so, there is a need for directing health education and weight control interventions at maternity care units in low income neighbourhoods.

## Competing interests

The authors declare that they have no competing interests.

## Authors' contributions

ES outlined the design of the study, analysed the results, participated in the statistical analyses and drafted the manuscript. AH provided data and participated in the analyses of results. GA performed statistical analyses. MA participated in the design of the study and the drafting of manuscript. All authors read and approved the final manuscript.

## Pre-publication history

The pre-publication history for this paper can be accessed here:



## Supplementary Material

Additional file 1 **Multilevel logistic regression of predictors of obesity in women in early pregnancy**. Data provided represent multilevel logistic regression analyses of predictors of obesity in women in early pregnancy. Two-level random intercept models. Click here for file
