# Supplementary material for: Obesity prevalence in a cohort of women in early pregnancy from a neighbourhood perspective
Source: BMC Pregnancy Childbirth. 2009 Aug 25;9:37. doi: 10.1186/1471-2393-9-37 (PMC2744903; doi:10.1186/1471-2393-9-37)
Supplement: Additional file 1 — Multilevel logistic regression of predictors of obesity in women in early pregnancy. Data provided represent multilevel logistic regression analyses of predictors of obesity in women in early pregnancy. Two-level random intercept models. [file 1471-2393-9-37-S1.doc]

**Additional file 1.**

**Format: DOC**

**Title: Multilevel logistic regression of predictors of obesity in women in early pregnancy**

**Description: Data provided represent multilevel logistic regression analyses of predictors of obesity in women in early pregnancy. Two-level random intercept models.**

|  | MODEL 1  Base model |  | MODEL 2  Individual model | | MODEL 3  Neighbourhood model | | MODEL 4  Individual and neighbourhood model | |
| --- | --- | --- | --- | --- | --- | --- | --- | --- |
|  | Beta value (SE) | Odds ratio (95% CI) | Beta value (SE) | Odds ratio  (95% CI) | Beta value (SE) | Odds ratio  (95% CI) | Beta value (SE) | Odds ratio  (95% CI) |
| **Intercept** | -2.924 (0.027) |  | -3.800 (0.054) |  | -3.447 (0.071) |  | -4.133 (0.081) |  |
| ***Fixed effects*** | | | | | | | | |
| **Year of pregnancy** | 0.079 (0.005) | 1.08 (1.07;1.09) | 0.089 (0.005) | 1.09 (1.08; 1.10) | 0.078 (0.005) | 1.08 (1.07; 1.09) | 0.089 (0.005) | 1.09 (1.08; 1.10) |
| **Age**  20-34 yrs (reference)  35 yrs or older |  |  | -  0.358 (0.050) | 1.00  1.43 (1.30; 2,26) |  |  | -  0.358 (0.051) | 1.00  1.43 (1.29; 1.58) |
| **Education**  Elementary  Secondary (2 yrs)  Secondary (3-4 yrs)  University (reference) |  |  | 1.083 (0.066)  0.807 (0.049)  0.376 (0.058)  - | 2.95 (2.60; 3.36)  2.24 (2.04; 2.47)  1.46 (1.30; 1.63)  1.00 |  |  | 1.050 (0.066)  0.791 (0.050)  0.367 (0.058)  - | 2.86 (2.51; 3.25)  2.21 (2.00; 2.43)  1.44 (1.29; 1.62)  1.00 |
| **Income**  1^st^ quintile (lowest)  2^nd^ quintile  3^rd^ quintile  4^th^ quintile  5^th^ quintile (reference) |  |  | 0.211 (0.065)  0.312 (0.057)  0.259 (0.049)  0.224 (0.042)  - | 1.24 (1.09; 1.40)  1.37 (1.22; 1.53)  1.30 (1.18; 1.43)  1.25 (1.15; 1.36)  1.00 |  |  | 0.167 (0.066)  0.278 (0.058)  0.230 (0.049)  0.204 (0.043)  - | 1.18 (1.04; 1.35)  1.32 (1.18; 1.48)  1.26 (1.14; 1.39)  1.23 (1.13; 1.33)  1.00 |
| **Neighbourhood economic status**  Resourceful (reference)  Medium level  Limited resources |  |  |  |  | -  0.470 (0.075)  0.812 (0.084) | 1.00  1.60 (1.38; 1.85)  2.25 (1.91; 2.66) | -  0.333 (0.073)  0.590 (0.082) | 1.00  1.40 (1.21; 1.61)  1.80 (1.54; 2.12) |
| ***Fixed effects*** | | | | | | | | |
| Intercept variance; neighbourhoods (SE) | 0.249 (0.024) |  | 0.162 (0.019) |  | 0.184 (0.020) |  | 0.134 (0.017) |  |
| Variance partition coefficient | 7.04% |  |  |  |  |  |  |  |
| Reduction of intercept variance |  |  | 32.9 % |  | 26.1% |  | 46.2 % |  |
